# Platelet Activation in Human Immunodeficiency Virus Type-1 Patients Is Not Altered with Cocaine Abuse

**DOI:** 10.1371/journal.pone.0130061

**Published:** 2015-06-15

**Authors:** Michelle Kiebala, Meera V. Singh, Michael S. Piepenbrink, Xing Qiu, James J. Kobie, Sanjay B. Maggirwar

**Affiliations:** 1 Department of Microbiology and Immunology, University of Rochester School of Medicine and Dentistry, Rochester, New York, United States of America; 2 Division of Infectious Diseases, University of Rochester School of Medicine and Dentistry, Rochester, New York, United States of America; 3 Department of Biostatistics and Computational Biology, University of Rochester School of Medicine and Dentistry, Rochester, New York, United States of America; University of South Carolina School of Medicine, UNITED STATES

## Abstract

Recent work has indicated that platelets, which are anucleate blood cells, significantly contribute to inflammatory disorders. Importantly, platelets also likely contribute to various inflammatory secondary disorders that are increasingly associated with Human Immunodeficiency Virus Type-1 (HIV) infection including neurological impairments and cardiovascular complications. Indeed, HIV infection is often associated with increased levels of platelet activators. Additionally, cocaine, a drug commonly abused by HIV-infected individuals, leads to increased platelet activation in humans. Considering that orchestrated signaling mechanisms are essential for platelet activation, and that nuclear factor-kappa B (NF-κB) inhibitors can alter platelet function, the role of NF-κB signaling in platelet activation during HIV infection warrants further investigation. Here we tested the hypothesis that inhibitory kappa B kinase complex (IKK) activation would be central for platelet activation induced by HIV and cocaine. Whole blood from HIV-positive and HIV-negative individuals, with or without cocaine abuse was used to assess platelet activation via flow cytometry whereas IKK activation was analyzed by performing immunoblotting and *in vitro* kinase assays. We demonstrate that increased platelet activation in HIV patients, as measured by CD62P expression, is not altered with reported cocaine use. Furthermore, cocaine and HIV do not activate platelets in whole blood when treated *ex vivo*. Finally, HIV-induced platelet activation does not involve the NF-κB signaling intermediate, IKKβ. Platelet activation in HIV patients is not altered with cocaine abuse. These results support the notion that non-IKK targeting approaches will be better suited for the treatment of HIV-associated inflammatory disorders.

## Introduction

The increased incidence of inflammatory secondary disorders associated with Human Immunodeficiency Virus Type-1 (HIV) infection, despite excellent control of the virus with combination antiretroviral therapy (cART), is an emerging health concern. Indeed, up to 50% of HIV-infected individuals will suffer from some form of HIV-associated neurological disorders [1], and there is a much higher incidence of atherosclerosis within the HIV-infected population [2, 3]. These inflammatory complications are further exacerbated by illegal drug use among HIV-infected individuals [4, 5]. Platelets, which are anucleate blood cells that are important for thrombosis, have also been shown to contribute to inflammatory processes through multiple mechanisms.

HIV infection is often associated with impaired platelet function, accumulation of platelet activators [6, 7], and increased platelet activation [8–12], regardless of cART [11, 13]. Platelet activation is also central to the neurological complications associated with HIV infection. Importantly, several large clinical studies have suggested the influence of platelets on the outcome of neurological impairment associated with HIV infection [14–16]. The above-mentioned mechanisms by which platelets contribute to inflammation are also important during the development of atherosclerosis [17].

Recent reports have demonstrated that platelets contain transcription factors, including nuclear factor-kappa B (NF-κB) family members and that NF-κB inhibitors can alter platelet function [18–22]. As platelets lack a nucleus, these studies suggest a non-transcriptional, yet critical role for NF-κB in platelets. Indeed, Karim et al. implicated inhibitory kappa B kinase beta (IKKβ), a kinase that regulates activation of NF-κB, in controlling platelet secretion through phosphorylation of SNAP-23, which is a target membrane SNARE (soluble *N*-ethylmaleimide-sensitive-factor (NSF) attachment protein receptor) protein important for the fusion of granules or endosomes with plasma membranes, a process necessary for cargo release [23]. Granule release is particularly important during platelet activation and facilitates their ability to mediate inflammation.

Drug abuse is a common co-morbidity associated with HIV infection. Cocaine abuse alone is well known to have multiple adverse effects on the cardiovascular system and the central nervous system (CNS) [24–26], and cocaine use is relatively common within the HIV-infected population [27]. Platelets express both dopamine receptors [28] and the dopamine transporter (DAT) [29]. Cocaine use, by itself, is well known to increase platelet activation *in vivo* as measured by an increase in platelet releasates and an increase in platelet aggregation [30–33], although cocaine alone may or may not activate platelets *in vitro* [32, 33]. Despite the interactions between both HIV and platelets, and cocaine and platelets, as well as the combined detrimental effects of these elements [4, 5], little is known about the activation state of platelets in HIV-infected individuals that also abuse cocaine.

Based on the ability of HIV and cocaine separately to enhance platelet activation, and the importance of the NF-κB pathway for platelet function, we hypothesized that IKK activation would be central for activation of platelets by HIV and cocaine. However, herein we demonstrate that patients with HIV infection and with reported cocaine use do not have increased platelet activation compared to their HIV-infected counterparts without reported cocaine use. Furthermore, we show that HIV-induced platelet activation does not involve IKKβ. A better understanding of the signaling events within platelets during HIV infection may prove useful for the development of new therapies targeting platelets in HIV-associated inflammatory secondary disorders.

## Materials and Methods

### Ethics Statement

All experiments involving the use of laboratory animals were carried out in accordance with the Animal Welfare Act and NIH guidelines. The animal protocol was approved by the University Committee on Animal Resources of the University of Rochester School of Medicine and Dentistry (URSMD) (Protocol number: UCAR-2013-008). The URSMD Vivarium and Division of Laboratory Animal Medicine are accredited by the Association for the Assessment and Accreditation of Laboratory Animal Care International. Strain C57BL/6 mice were purchased from The Jackson Laboratory (Bar Harbor, ME, USA). All efforts were made to minimize suffering.

Whole blood was obtained from adult male and female donors, and all patients gave written informed consent for all procedures, in accordance with the Declaration of Helinski, which were approved by the UR Research Subjects Review Board (Protocol numbers: RSRB00044059, RSRB00044074).

### Reagents

Cocaine-HCl was purchased from Sigma-Aldrich (St. Louis, MO, USA). HIV Tat 1–72 was obtained from Philip Ray (University of Kentucky, Lexington, KY, USA), and was used at 100nM (~800ng/mL) concentration, as described [34]. Adenosine diphosphate (ADP) was purchased from Chrono-log (Havertown, PA, USA). ACK red blood cell (RBC) lysis buffer was purchased from Invitrogen/Life Sciences (Grand Island, NY, USA).

### Patient samples

Blood samples were collected from HIV-negative, healthy controls (N = 61), HIV-positive individuals without cocaine use (N = 37), and HIV-positive individuals with reported cocaine use within the previous one year (N = 16). All HIV-positive individuals were on antiretroviral therapy at the time of the blood draw. Blood was drawn into acid citrate dextrose (ACD)–buffered vacutainer tubes (BD Biosciences, San Jose, CA). 100μL blood per sample was fixed and stained as described [11, 13]. Patient demographics are described in Table 1.

**Table 1:** Demographic and clinical characteristics of study participants.

Characteristics	HIV-	HIV+	HIV+Coc+
Mean age +/- SD, y	35 +/- 13	46 +/- 14	52 +/- 5
Race, *N*, (%)			
Black	8 (13)	19 (51)	10 (63)
White	48 (79)	12 (32)	4 (25)
Hispanic	1 (2)	5 (14)	2 (13)
Asian	2 (3)	0 (0)	0 (0)
Unspecified	2 (3)	1 (3)	0 (0)
Gender, *N* (%)			
Male	32 (52)	29 (78)	13 (81)
Female	29 (48)	8 (22)	3 (19)
Mean CD4 count +/- SD, *Cells/mm* ^*3*^	NA	556 +/- 248	563 +/- 257
Viral Load			
Undetectable, *N* (%)	NA	22 (59)	8 (50)
Detectable, Mean +/- SD, *RNA copies/mL*	NA	145 +/- 159	65 +/- 46

HIV-negative, healthy controls (HIV-, N = 61), HIV-positive individuals without cocaine use (HIV+Coc-, N = 37), and HIV-positive individuals with reported cocaine use within the previous one year (HIV+Coc+, N = 16) were enrolled in the study. All HIV-positive individuals were on antiretroviral therapy at the time of the blood draw. Unless otherwise stated, *N* indicates sample number with percentage of total sample group in parentheses. NA indicates not applicable.

### Cocaine treatment in mice

Wild-type C57BL/6 mice were injected intraperitoneally (IP) with phosphate buffered saline (PBS), 5mg/kg cocaine, or 50mg/kg cocaine three times per week for three weeks. Following the final injection, blood was collected via submandibular bleed into tubes containing ACD, and platelet activation in whole blood was assessed via CD62P staining.

### Platelet activation assay

Platelet activation was assessed in human whole blood via CD62P (P-selectin) surface expression, using a previously described method [11, 13], within 1h of the blood draw. Similarly, platelet activation in mouse blood was assessed using 2.5μL anti-mouse CD61-PE (catalog # 561910) and 1μL anti-mouse CD62P-FITC (catalog # 561923; BD Biosciences, San Jose, CA).

### Production of infectious HIV

Virus stocks were generated by transfecting human embryonic kidney 293T cells with pNLENG1 IRES construct (X4 tropic pNL4-3 backbone, kind gift from Dr. David Levy, New York University) using polyethylenimine (PEI, Sigma Aldrich, St. Louis, MO, USA). Virus was concentrated using PEG6000 as described [35], and resuspended in PBS. The p24 concentration was determined by ELISA (ABL Biosciences, Rockville, MD, USA).

### Human platelet isolation

Whole blood was sequentially centrifuged and cells were washed to collect a purified platelet concentrate as described [36, 37]. Platelet purity was determined to be **>** 99%.

### Immunoblot analysis

Platelet whole cell lysates were prepared in ELB buffer and immunoblot analysis was performed as described [38]. The following primary antibodies were used: IKKα (sc-7218), IKKβ (sc-8014), IκBα (sc-371), and α-Tubulin (sc-8035; 1:1000; Santa Cruz Biotechnologies Inc., Santa Cruz, CA, USA).

### Electrophoretic mobility shift assays

Platelet lysates were prepared in ELB buffer as described above. Electrophoretic mobility shift assays (EMSA) were performed using platelet lysates as described [38].

### IKKβ in vitro kinase assay

Platelet lysates were prepared and immunoprecipitation was performed with antibodies specific to IKKβ (sc-8014; Santa Cruz Biotechnologies Inc., Santa Cruz, CA, USA). Immune complexes were incubated with substrate, recombinant IκBα (rIκBα), and 10mCi [γ-^32^P]ATP. IKK activity was measured by the incorporation of [^32^P] at the IKK-sensitive site of the rIκBα substrate via densitometric analysis of autoradiograms (ImageJ software; NIH, Bethesda, MD.)

### Statistical analyses

**Fig 1:**
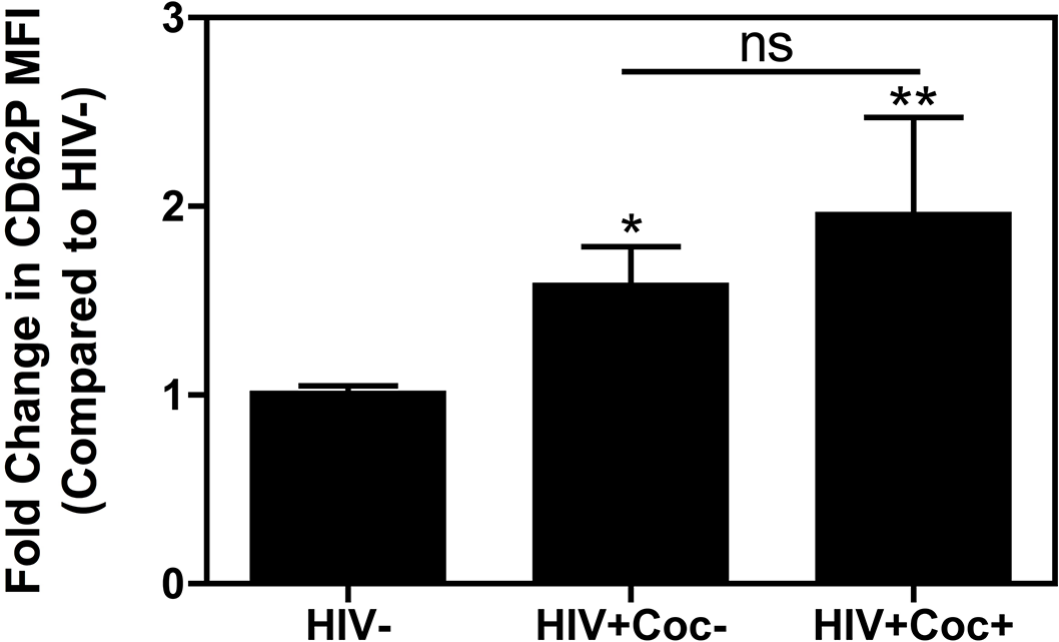
Increased platelet activation in HIV patients is not altered with reported cocaine use. Blood samples were collected from HIV-negative, healthy subjects (HIV-, N = 61), HIV-positive individuals without cocaine use (HIV+Coc-, N = 37), and HIV-positive individuals with reported cocaine use within one year (HIV+Coc+, N = 16), and were fixed and stained as described in the methods section. Platelet CD62P expression was significantly higher in HIV+Coc- and HIV+Coc+ samples as compared to HIV- subjects (* p = 0.038, ** p = 0.008, respectively). CD62P expression levels between HIV+Coc- and HIV+Coc+ samples were statistically similar (ns, p = 0.492). Data are represented as fold change in CD62P MFI as compared to HIV- subjects and are shown as mean ± SEM.

For the analysis of patient data shown in Fig 1, Tukey’s *post hoc* analysis (R 3.0.2, R Foundation for Statistical Computing, Vienna, Austria) was used to test whether there are significant mean differences between three groups.

For all other analyses involving multiple sample groups, statistical significance was determined using one-way ANOVA followed by Bonferroni’s test for multiple comparisons (Prism 4.0 software; GraphPad Software, La Jolla, CA, USA).

## Results

### Elevated platelet activation during HIV infection is not further heightened with reported cocaine use

As HIV-infected patients are experiencing much longer life spans due to the success of antiretroviral therapy, consequently there is an increased incidence of HIV-related inflammatory secondary disorders in this population, including neurological and cardiovascular complications [1–3]. These inflammatory disorders are often exacerbated by common co-morbidities, such as illegal drug use. In particular, cocaine use is known to complicate both neurological and cardiovascular HIV-related disorders [4, 5]. Through the release of their granular contents, platelets are important inflammatory mediators [39, 40], and both HIV and cocaine, separately, can induce platelet activation [8–12, 30–33]. However, the combined effect of HIV and cocaine together on platelet activation is less known. Based on this, we sought to measure evidence of platelet activation in healthy, HIV-negative subjects (HIV-, N = 61), HIV-positive individuals without cocaine use (HIV+Coc-, N = 37), and HIV-positive individuals with reported cocaine use within one year of the blood draw (HIV+Coc+, N = 16). Significant mean differences between these three groups were detected by one-way ANOVA *F*-test (p = 0.003). Tukey’s *post hoc* analysis revealed a significantly higher degree of platelet activation in the HIV+Coc- subjects as compared to the HIV- individuals (p = 0.038, Fig 1), which is consistent with previous results [11]. There was also increased platelet activation in the HIV+Coc+ patients as compared to the HIV- individuals (p = 0.008, Fig 1). Interestingly, platelet activity in the HIV+Coc+ group as compared to the HIV+Coc- group was found to be statistically similar (p = 0.492, Fig 1). This suggests that although HIV and cocaine alone can affect platelets, there is not a combined, additive effect of HIV and cocaine on platelet activation, likely indicating that both stimulate platelets via a similar mechanism.

### Cocaine and HIV do not activate platelets in whole blood when treated ex vivo

**Fig 2:**
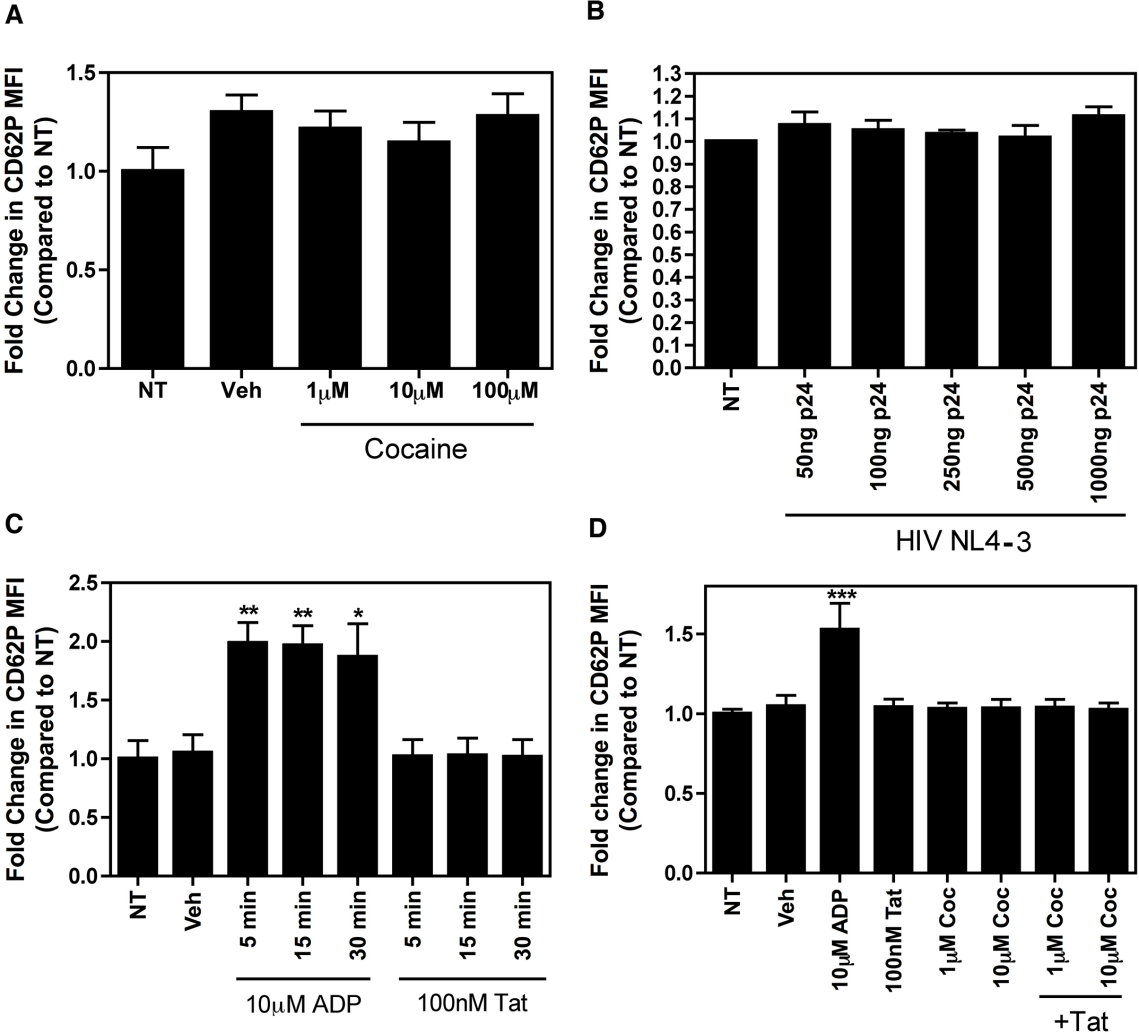
Cocaine and HIV do not activate platelets in whole blood when treated *ex vivo*. Whole blood from healthy subjects (3 to 4 donors) was left untreated (NT) or was treated with cocaine (A), infectious NL4-3 HIV (B), ADP or Tat (C) (D), for 30, 60, and 30 minutes respectively, or as indicated, following which CD62P expression was assessed as a marker of platelet activation via flow cytometry. Treatment with cocaine, HIV, and Tat did not result in increased platelet activation. In each experiment, as indicated, treatment with 1 μL dH_2_O for 30 minutes was used as a vehicle (Veh) control. Data are represented as fold change in CD62P MFI as compared to NT samples and are shown as mean ± SEM. * p<0.05, ** p<0.01, *** p<0.001.

Based on previous reports of elevated platelet activation in individuals with HIV infection and/or cocaine abuse [8–11, 13, 33], we next sought to determine the direct effects of cocaine, HIV, and the HIV protein Tat on platelet activation, *ex vivo*. To do this, whole blood from healthy individuals (N = 4) was treated with 1, 10, and 100μM cocaine for 30 minutes. Treatment with 1μL dH_2_O was used as a vehicle control. We chose these concentrations based on reported plasma levels of cocaine following drug administration, which range from 0.3 to 7μM depending on the route of administration and whether venous or arterial levels were measured [41]. Following treatment, platelet activation in whole blood was assessed via staining for CD62P. Our results indicated that the administration of cocaine in whole blood failed to activate platelets (Fig 2). Furthermore, as expected [32], a physiologically irrelevant, excessive amount of cocaine (10mM) was able to stimulate platelets (S1 Fig). Failure of cocaine to activate platelets in these assays was not due to our approach of treating platelets in whole blood, as cocaine also did not induce platelet activation when added to isolated platelets (S2 Fig).

Next, we incubated whole blood from healthy individuals (N = 3) with increasing amounts of infectious X4-tropic HIV virus particles for 1 hour, following which we measured platelet activation. HIV virions are capable of binding to platelets via attachment factors on the surface of platelets including CXCR4, DC-SIGN, and CLEC-2 [42–45], hence the use of X4-tropic virus was important. As shown in Fig 2, infectious HIV virions did not induce platelet activation. However, as viral load is often below the limit of detection in HIV-positive patients receiving antiretroviral therapy, it is unlikely that platelets in circulation would be exposed to infectious virions in these patients. In contrast, low levels of soluble viral proteins, including Tat, are persistently released from infected cells despite sufficient control of viral replication [46–48]. For this reason, we next assessed the effect of the HIV protein Tat on platelet activation. Following exposure of whole blood from healthy individuals (N = 3) to Tat for 5, 15, or 30 minutes, there was no detectable increase in platelet activation (Fig 2). Under the same conditions, addition of the mild platelet agonist ADP (positive control) resulted in significant platelet activation (Fig 2). Although a single report has demonstrated that Tat can activate platelets *in vitro* [49], a lack of a direct effect of Tat on platelets is consistent with previous work from our laboratory showing that Tat protein did not cause activation of isolated human platelets [7]. We also aimed to determine the combined effect of Tat and cocaine on platelet activation. Exposure of whole blood from healthy individuals (N = 3) to 1 and 10μM cocaine for 5 minutes followed by addition of Tat for 30 minutes, did not result in increased platelet activation, whereas under similar conditions, exposure to ADP and cocaine for 30 minutes did activate platelets (Fig 2). These results suggest that neither cocaine, at physiologically relevant concentrations, infectious HIV, nor the viral protein Tat can activate platelets when added *ex vivo* to human blood.

### In vivo evidence that cocaine leads to platelet activation

**Fig 3:**
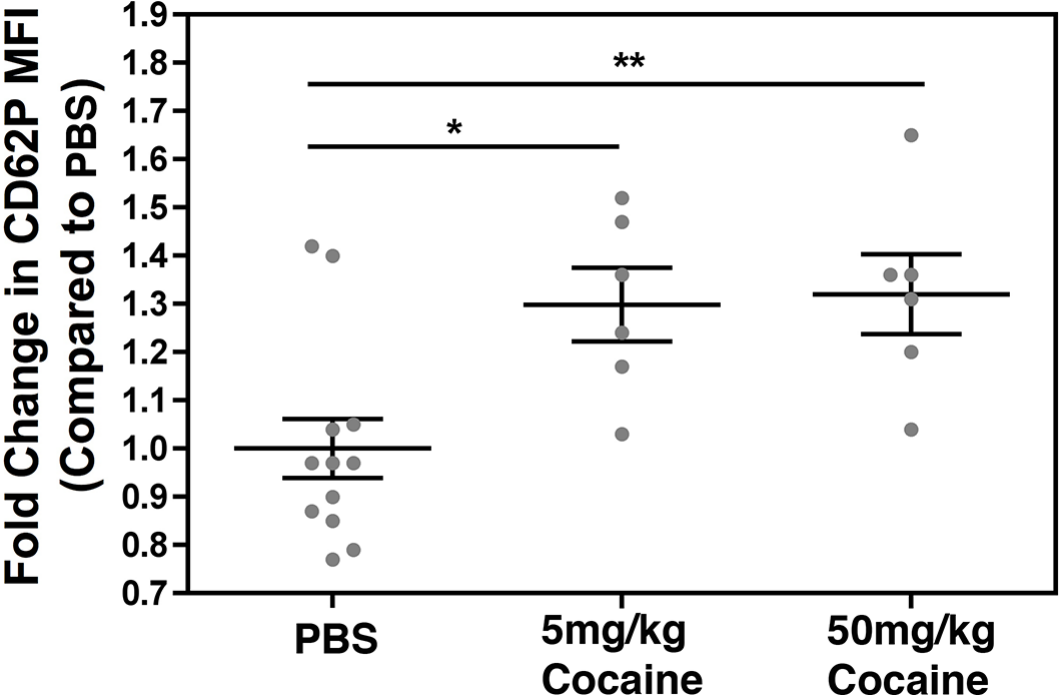
Cocaine indirectly activates platelets *in vivo*. Wild-type C57BL/6 mice were injected intraperitoneally (IP) with either PBS (N = 12), 5mg/kg cocaine (N = 6), or 50mg/kg cocaine (N = 6) three times per week for three weeks. Following the final injection, blood was collected into tubes containing acid citrate dextrose, and platelet activation in whole blood was assessed via CD62P staining. Treatment with both concentrations of cocaine resulted in increased platelet activation as compared to the PBS treated mice (* p<0.05, ** p<0.01). Data are represented as fold change in CD62P MFI as compared to PBS treated samples and are shown as mean ± SEM.

Although we did not see evidence of Tat-induced platelet activation in whole blood treated *ex vivo*, our group has previously shown that Tat does activate platelets in mice [50]. In addition, a recent study reported increased platelet activation in mice 30 minutes after IP injection with 30mg/kg cocaine [51]. Hence, we next sought to determine platelet activation levels in mice following cocaine injection three times per week for three weeks, as a model of chronic exposure to the drug, which is often the case with cocaine users. In this model both 5mg/kg (N = 6) and 50mg/kg (N = 6) cocaine, but not PBS (N = 12) administered on the same schedule, stimulated platelets (Fig 3). In sum, both Tat and cocaine can stimulate platelets *in vivo*, albeit via indirect mechanisms.

### NF-κB signaling mechanism not involved in HIV-induced platelet activation

**Fig 4:**
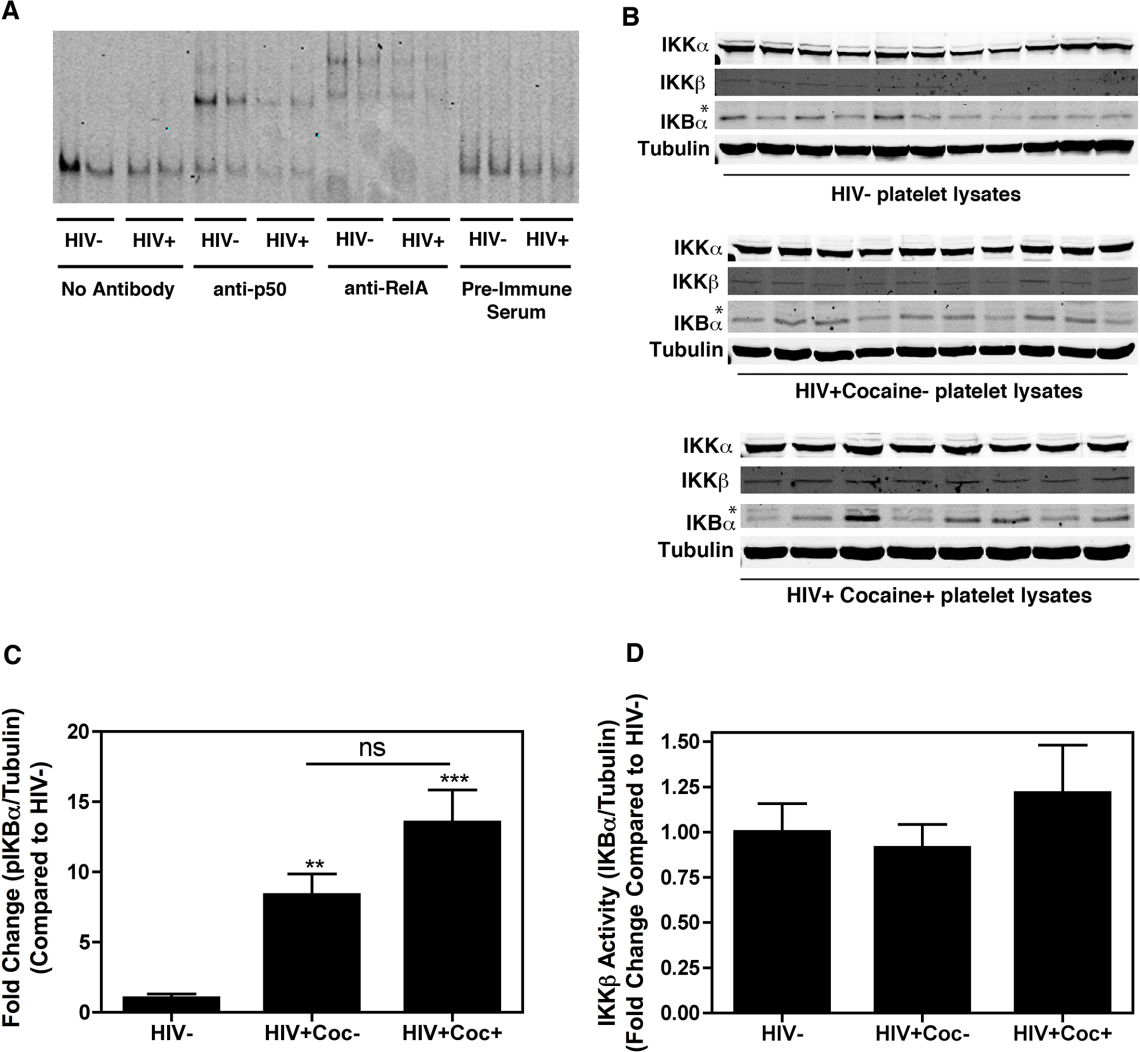
HIV-induced platelet activation does not involve NF-κB signaling mechanism. (A) Platelet lysates collected from HIV-negative subjects (HIV-, N = 2) and HIV-positive subjects (HIV+, N = 2) were subjected to electrophoretic mobility shift assays followed by supershift with anti-p50 and anti-RelA antibodies. Incubation with pre-immune serum was included as a control. Both p50 and RelA antibodies altered the mobility of bands, suggesting the presence of these two molecules in NF-κB/DNA complexes, however, no apparent differences were found between HIV- and HIV+ samples. (B) Platelet lysates collected from HIV-negative, healthy subjects (HIV-, N = 11), HIV-positive patients without cocaine use (HIV+Cocaine-, N = 10), and HIV-positive patients with reported cocaine use within one year (HIV+Cocaine+, N = 8) were subjected to immunoblot analysis with antibodies against IKKα, IKKβ, IκBα, or α-Tubulin. Expression levels of IKKα and IKKβ were noted to be similar between the groups. There was an increase in IκBα phosphorylation as evidenced by the appearance of a less mobile, upper band (*), which was confirmed by densitometric analysis of this upper phospho-IκBα band as shown in (C). (D) Platelet lysates collected from HIV- subjects (N = 15), HIV+Coc- (N = 10), and HIV+Coc+ (N = 5) were subjected to immunoprecipitation with an anti-IKKβ antibody followed by an IKKβ *in vitro* kinase assay. Incorporation of [^32^P] into the recombinant IκBα substrate was measured by densitometric analysis of autoradiograms. No significant differences in IKKβ activity between the sample groups were noted. Data are represented as fold change in IKKβ activity compared to the HIV- samples and are shown as mean ± SEM.

Although the DNA binding activity of NF-κB proteins may not be essential for their roles in platelets, it can serve as a measure of activation of this signaling mechanism. Thus, we first determined whether there were apparent differences in the DNA binding capabilities of NF-κB proteins in platelets isolated from healthy individuals (HIV-, N = 2) and from HIV-positive subjects (HIV+, N = 2) using EMSA. As shown in Fig 4, platelet-derived NF-κB retained its’ DNA binding ability with little difference between the HIV+ and HIV- samples. In addition, incubation with antibodies against NF-κB family members p50 and RelA altered the mobility of bands, indicating the presence of these two molecules in NF-κB/DNA complexes (Fig 4). Absence of a band shift following incubation with cRel, RelB, and p52 antibodies suggested that these molecules were not present in the complexes (S3 Fig).

Within the NF-κB signaling pathway, IKK represents a critical regulatory hub where signals from multiple upstream pathways converge, and are then dispersed downstream toward NF-κB transcriptional activation [52–55]. The IKK complex is comprised of IKKα and IKKβ, both of which have kinase activity, and IKKγ, which has structural and regulatory functions. Based on the importance of the IKK complex within NF-κB signaling, and because of the non-transcriptional role of NF-κB in platelets, we hypothesized that IKK would be important for HIV-induced platelet activation. Although the IKK complex has the capacity to phosphorylate many downstream targets, its main target within the NF-κB pathway is the inhibitory kappa B protein (IκBα). We first assessed expression levels of IKKα, IKKβ, and IκBα via immunoblot analysis in platelet lysates isolated from HIV- individuals (N = 11), HIV+Coc- patients (N = 10), and HIV+Coc+ patients (N = 8). While there were no clear differences in IKKα and IKKβ expression levels between the groups, there did appear to be increased presence of a slightly higher molecular weight band in the IκBα blots in the HIV+Coc- and HIV+Coc+ groups (as marked by an asterisk in Fig 4). This less mobile band indicates IκBα phosphorylation, which is an indirect measure of IKK activity. Densitometric analysis of this upper phospho-IκBα band confirmed that there was increased IκBα phosphorylation in both groups compared to the HIV- group, whereas although there was a trend for increased IκBα phosphorylation between the HIV+Coc- group and the HIV+Coc+ group, this difference was not significant (Fig 4). As a more direct measure of IKK activity, we next performed IKKβ *in vitro* kinase assays where we immunoprecipitated IKKβ from platelets isolated from HIV- (N = 15), HIV+Coc- (N = 10), and HIV+Coc+ (N = 5) individuals, and measured its ability to incorporate [^32^P] in the recombinant IκBα molecule (substrate). As shown in Fig 4, there was no change in IKKβ activity levels between the groups. As we did see increased levels of IκBα phosphorylation via immunoblot analysis (Fig 4), without increased IKKβ activity levels via *in vitro* kinase assay (Fig 4), this suggests that other kinases which may be closely associated with IKK are involved in IκBα phosphorylation within platelets in HIV+ patients. Overall, our data suggest that the NF-κB signaling mechanism, including IKKβ, is not involved in HIV-induced platelet activation, with or without cocaine use.

### Platelets from HIV-positive patients are sensitized to activation by ADP plus cocaine

**Fig 5:**
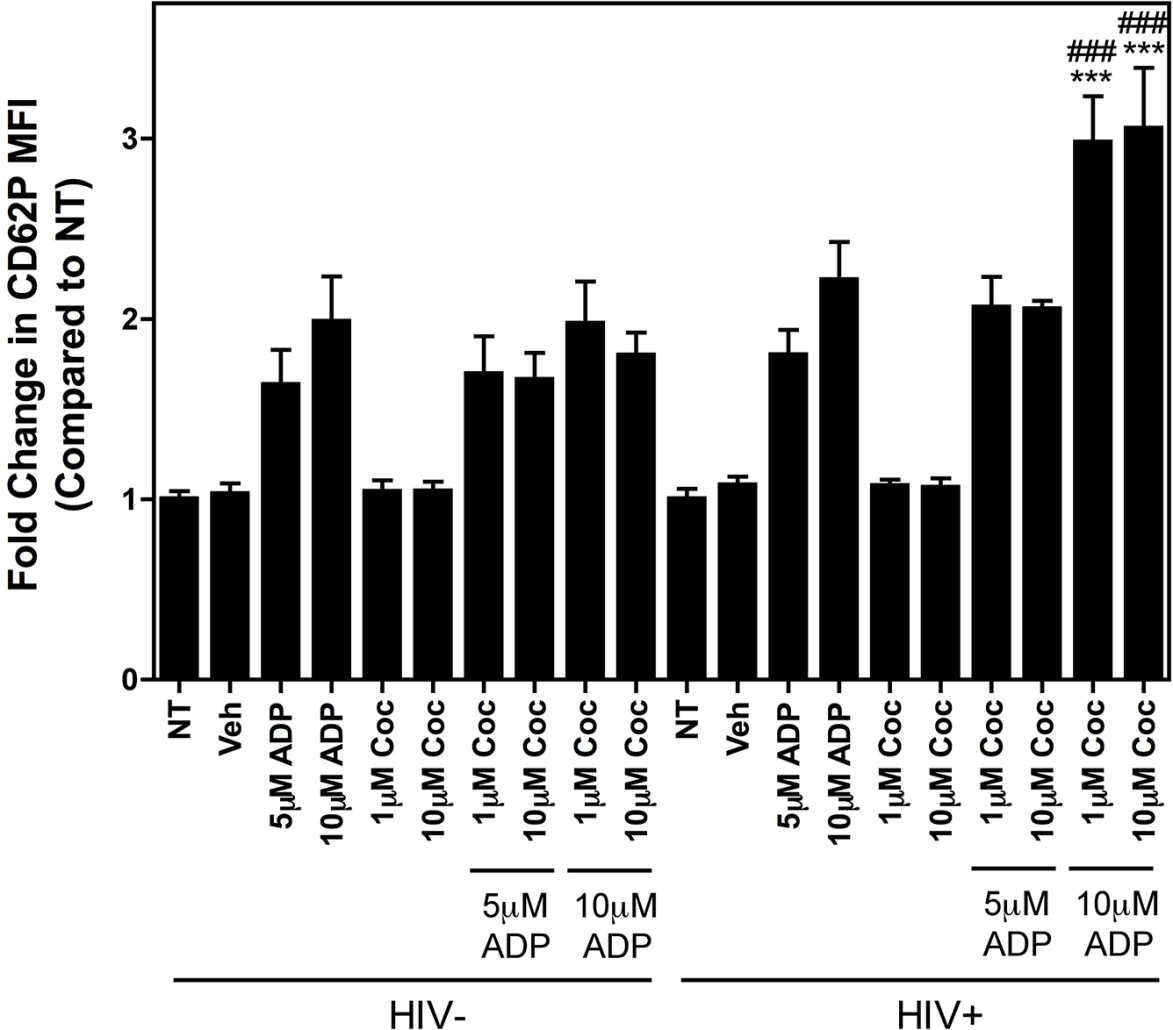
Cocaine sensitizes platelets from HIV+, but not HIV-, donors to activation by ADP. Whole blood from healthy subjects (HIV-, N = 4) or HIV-positive subjects (HIV+, N = 4) was either left untreated (NT) or was treated with the indicated concentrations of cocaine alone or together with the indicated concentrations of ADP for 30 minutes. Platelet activation in whole blood was assessed via CD62P staining. In the HIV+ samples there was a significant increase in platelet activation following treatment with 10 μM ADP plus cocaine (*** p<0.001 compared to 10 μM ADP alone in the HIV+ samples, ^###^ p<0.001 compared to 10 μM ADP plus cocaine in the HIV- samples). Treatment with 1 μL DMSO for 5 minutes followed by 1 μL dH_2_O for 30 minutes was used as a vehicle (Veh) control. Data are represented as fold change in CD62P MFI as compared to NT samples and are shown as mean ± SEM.

It is apparent that HIV+ individuals that use cocaine have an increased risk of the neurological and thrombotic inflammatory secondary disorders associated with HIV infection, as compared to their non-drug using, HIV-infected counterparts [4, 5]. Although we did not see enhanced platelet activation in HIV+Coc+ subjects as compared to HIV+Coc- patients, we hypothesized that platelets in HIV+ individuals might be sensitized to activation by platelet agonist plus cocaine. Whole blood from HIV- subjects (N = 4) and from HIV+ individuals (N = 4) was pre-treated with 1 and 10μM cocaine for 5 minutes followed by 30 minutes treatment with 5 and 10μM ADP. Treatment with ADP alone in both HIV- and HIV+ blood samples resulted in platelet activation, whereas treatment with cocaine alone did not (Fig 5). In the HIV- samples ADP plus cocaine failed to activate platelets beyond the level exerted by ADP alone, however, in the HIV+ samples, ADP (10μM) plus two different doses of cocaine readily triggered platelet activity as compared to ADP alone in the HIV+ samples, and that of ADP plus cocaine in the HIV- samples (Fig 5). Collectively, our results suggest that HIV and cocaine may trigger platelet activity indirectly via specific stimuli.

## Discussion

HIV clearly has an effect on platelet activation as many studies have shown an increase in markers of platelet activation in HIV-infected individuals. However, despite expression of HIV attachment factors on the surface of platelets [42–45], the effect of HIV on platelets is likely indirect. This is not unexpected considering that platelets are anucleate, as such HIV does not productively infect platelets [56]. The indirect effect of HIV on platelet activation is also supported by our data showing a lack of platelet activation following incubation of whole blood with infectious HIV. Likewise, cocaine also indirectly affects platelet activation. Cocaine users have increased markers of platelet activation immediately following cocaine use, however, the data shown herein, as well as data from other labs [33], has shown that incubation of isolated platelets with cocaine does not induce platelet activation. This lack of a platelet response to cocaine when treated in whole blood is unlikely due to the presence of the anticoagulant, ACD, in the blood, as platelets in whole blood collected in this manner are responsive to other platelet agonists, and as isolated platelets, which are removed from the anticoagulant, are also unresponsive to cocaine (S2 Fig). Since we show here that there is not a combined, additive effect of HIV and cocaine on platelet activation, this likely indicates that both affect platelet activation via a similar mechanism. Based on previous work from our group implicating the importance of CD40 ligand signaling in endothelial cells for Tat-induced blood brain barrier permeability [50], we speculate that HIV and cocaine influence platelet activation *in vivo* through either changes in endothelial cell activation or gross changes in the vasculature, such as altered contractility or flow dynamics. Further research is needed to delineate how HIV and cocaine may act in combination to alter the vasculature.

While it is true that aberrant platelet activation appears to contribute significantly to the chronic inflammation associated with HIV infection, a low level of platelet activation may be beneficial in this context. Reports have shown that release of the CXC chemokine, CXCL4 (platelet factor 4, PF4), from platelet α-granules upon their activation is inhibitory to HIV replication [57, 58], thus suggesting a protective role for platelets against HIV infection. This, together with the importance of platelets for wound healing, highlights the fact that potential HIV adjunctive therapies targeting platelets must not completely block platelet activation.

Admittedly, as we assessed cocaine use by self-report we may have underestimated the combined effect of HIV and cocaine on platelet activation. It is also possible that the large variation in platelet activation in the HIV+ cocaine+ group was due to different amounts and frequency of cocaine use among study participants. Our protocol assessed positive versus negative cocaine use within the past one year, rather than a more detailed assessment of drug use habits. Nonetheless, our study supports the notion that both HIV and cocaine can independently influence platelet activation, and it is likely that cocaine enhances HIV-induced inflammation [25], especially with chronic use.

Dysregulation of signaling pathways, other than NF-κB, probably contribute to the increased platelet activity found in HIV-infected patients. Likewise, alterations in signaling pathways within platelets, such as p38 MAPK, are evident within individuals afflicted with other inflammatory diseases associated with increased risk of thrombotic complications, including diabetes mellitus [59, 60]. Considering the lack of adjunctive therapies to remedy HIV-associated inflammatory disorders, as well as the likely contribution of platelets to these disorders, a better understanding of the mechanisms involved in platelet activation induced by HIV and cocaine, may contribute to the development of novel therapies.

## Supporting Information

S1 Fig Physiologically irrelevant, excessive amount of cocaine does stimulate platelets in whole blood when treated *ex vivo*. Whole blood from healthy subjects (2 donors) was left untreated (NT) or was treated with 10mM cocaine for 30 minutes, following which CD62P expression was assessed as a marker of platelet activation via flow cytometry. Treatment with this excessive amount of cocaine did result in increased platelet activation. Data are represented as fold change in CD62P MFI as compared to NT samples and are shown as mean ± SEM.
(TIF)

S2 Fig Cocaine does not stimulate isolated platelets *in vitro*. Isolated platelets from healthy subjects (4 donors) were left untreated (NT) or were treated with the indicated concentrations of cocaine for 30 minutes, following which CD62P expression was assessed as a marker of platelet activation via flow cytometry. Treatment with cocaine did not result in increased platelet activation. Treatment with 1 μL dH_2_O for 30 minutes was used as a vehicle (Veh) control. Data are represented as fold change in CD62P MFI as compared to NT samples and are shown as mean ± SEM.
(TIF)

S3 Fig cRel, RelB, and p52 are not present in NF-κB/DNA complexes in platelets. Platelet lysates collected from HIV-negative subjects (HIV-, N = 2) and HIV-positive subjects (HIV+, N = 2) were subjected to electrophoretic mobility shift assays followed by supershift with anti-cRel, anti-RelB, and anti-p52 antibodies. These antibodies did not alter the mobility of bands, suggesting that these molecules are not present in NF-κB/DNA complexes in platelets. There were also no apparent differences between HIV- and HIV+ samples.
(TIF)

## References

[1] Heaton RK, Clifford DB, Franklin DR Jr., Woods SP, Ake C, Vaida F, et al HIV-associated neurocognitive disorders persist in the era of potent antiretroviral therapy: CHARTER Study. Neurology. 2010;75(23):2087–96. 10.1212/WNL.0b013e318200d727 21135382 PMC2995535

[2] Islam FM, Wu J, Jansson J, Wilson DP. Relative risk of cardiovascular disease among people living with HIV: a systematic review and meta-analysis. HIV Med. 2012;13(8):453–68. 10.1111/j.1468-1293.2012.00996.x 22413967

[3] Palella FJ Jr., Phair JP. Cardiovascular disease in HIV infection. Curr Opin HIV AIDS. 2011;6(4):266–71. 10.1097/COH.0b013e328347876c 21546831 PMC3501268

[4] Buch S, Yao H, Guo M, Mori T, Su TP, Wang J. Cocaine and HIV-1 interplay: molecular mechanisms of action and addiction. J Neuroimmune Pharmacol. 2011;6(4):503–15. 10.1007/s11481-011-9297-0 21766222 PMC3208732

[5] Mosunjac MI, Sundstrom JB, Heninger M, Ansari AA, Mosunjac MB. Combined pathological effects of cocaine abuse and HIV infection on the cardiovascular system: an autopsy study of 187 cases from the Fulton County Medical Examiner's office. Am J Forensic Med Pathol. 2008;29(1):9–13. 10.1097/PAF.0b013e318165152f 19749609

[6] Gelbard HA, Nottet HS, Swindells S, Jett M, Dzenko KA, Genis P, et al Platelet-activating factor: a candidate human immunodeficiency virus type 1-induced neurotoxin. J Virol. 1994;68(7):4628–35. 8207837 10.1128/jvi.68.7.4628-4635.1994 PMC236390

[7] Sui Z, Sniderhan LF, Schifitto G, Phipps RP, Gelbard HA, Dewhurst S, et al Functional synergy between CD40 ligand and HIV-1 Tat contributes to inflammation: implications in HIV type 1 dementia. J Immunol. 2007;178(5):3226–36. 17312171 10.4049/jimmunol.178.5.3226

[8] Corrales-Medina VF, Simkins J, Chirinos JA, Serpa JA, Horstman LL, Jy W, et al Increased levels of platelet microparticles in HIV-infected patients with good response to highly active antiretroviral therapy. J Acquir Immune Defic Syndr. 2010;54(2):217–8. 10.1097/QAI.0b013e3181c8f4c9 20505474

[9] Holme PA, Muller F, Solum NO, Brosstad F, Froland SS, Aukrust P. Enhanced activation of platelets with abnormal release of RANTES in human immunodeficiency virus type 1 infection. FASEB J. 1998;12(1):79–89. 9438413 10.1096/fasebj.12.1.79

[10] Satchell CS, Cotter AG, O'Connor EF, Peace AJ, Tedesco AF, Clare A, et al Platelet function and HIV: a case-control study. AIDS. 2010;24(5):649–57. 10.1097/QAD.0b013e328336098c 20177361

[11] Singh MV, Davidson DC, Jackson JW, Singh VB, Silva J, Ramirez SH, et al Characterization of platelet-monocyte complexes in HIV-1-infected individuals: possible role in HIV-associated neuroinflammation. J Immunol. 2014;192(10):4674–84. 10.4049/jimmunol.1302318 24729609 PMC4011982

[12] Gresele P, Falcinelli E, Sebastiano M, Baldelli F. Endothelial and platelet function alterations in HIV-infected patients. Thromb Res. 2012;129(3):301–8. 10.1016/j.thromres.2011.11.022 22192157

[13] Singh MV, Davidson DC, Kiebala M, Maggirwar SB. Detection of circulating platelet-monocyte complexes in persons infected with human immunodeficiency virus type-1. J Virol Methods. 2012;181(2):170–6. 10.1016/j.jviromet.2012.02.005 22387340 PMC3322263

[14] Ragin AB, D'Souza G, Reynolds S, Miller E, Sacktor N, Selnes OA, et al Platelet decline as a predictor of brain injury in HIV infection. J Neurovirol. 2011;17(5):487–95. 10.1007/s13365-011-0053-2 21956288 PMC3472427

[15] Wachtman LM, Tarwater PM, Queen SE, Adams RJ, Mankowski JL. Platelet decline: an early predictive hematologic marker of simian immunodeficiency virus central nervous system disease. J Neurovirol. 2006;12(1):25–33. 16595371 10.1080/13550280500516484

[16] Wachtman LM, Skolasky RL, Tarwater PM, Esposito D, Schifitto G, Marder K, et al Platelet decline: an avenue for investigation into the pathogenesis of human immunodeficiency virus-associated dementia. Arch Neurol. 2007;64(9):1264–72. 17846264 10.1001/archneur.64.9.1264

[17] Gawaz M, Langer H, May AE. Platelets in inflammation and atherogenesis. J Clin Invest. 2005;115(12):3378–84. 16322783 10.1172/JCI27196 PMC1297269

[18] Liu F, Morris S, Epps J, Carroll R. Demonstration of an activation regulated NF-kappaB/I-kappaBalpha complex in human platelets. Thromb Res. 2002;106(4–5):199–203. 12297126 10.1016/s0049-3848(02)00130-5

[19] Malaver E, Romaniuk MA, D'Atri LP, Pozner RG, Negrotto S, Benzadon R, et al NF-kappaB inhibitors impair platelet activation responses. J Thromb Haemost. 2009;7(8):1333–43. 10.1111/j.1538-7836.2009.03492.x 19566544

[20] Spinelli SL, Casey AE, Pollock SJ, Gertz JM, McMillan DH, Narasipura SD, et al Platelets and megakaryocytes contain functional nuclear factor-kappaB. Arterioscler Thromb Vasc Biol. 2010;30(3):591–8. 10.1161/ATVBAHA.109.197343 20042710 PMC2853005

[21] Hachem A, Yacoub D, Zaid Y, Mourad W, Merhi Y. Involvement of nuclear factor kappaB in platelet CD40 signaling. Biochem Biophys Res Commun. 2012;425(1):58–63. 10.1016/j.bbrc.2012.07.049 22820189

[22] Sahler J, Bernard JJ, Spinelli SL, Blumberg N, Phipps RP. The Feverfew plant-derived compound, parthenolide enhances platelet production and attenuates platelet activation through NF-kappaB inhibition. Thromb Res. 2011;127(5):426–34. 10.1016/j.thromres.2010.12.013 21272923 PMC3081947

[23] Karim ZA, Zhang J, Banerjee M, Chicka MC, Al Hawas R, Hamilton TR, et al IkappaB kinase phosphorylation of SNAP-23 controls platelet secretion. Blood. 2013;121(22):4567–74. 10.1182/blood-2012-11-470468 23613522 PMC3668489

[24] Afonso L, Mohammad T, Thatai D. Crack whips the heart: a review of the cardiovascular toxicity of cocaine. Am J Cardiol. 2007;100(6):1040–3. 17826394 10.1016/j.amjcard.2007.04.049

[25] Clark KH, Wiley CA, Bradberry CW. Psychostimulant abuse and neuroinflammation: emerging evidence of their interconnection. Neurotox Res. 2013;23(2):174–88. 10.1007/s12640-012-9334-7 22714667

[26] Maraj S, Figueredo VM, Lynn Morris D. Cocaine and the heart. Clin Cardiol. 2010;33(5):264–9. 10.1002/clc.20746 20513064 PMC6652820

[27] Klinkenberg WD, Sacks S. Mental disorders and drug abuse in persons living with HIV/AIDS. AIDS Care. 2004;16 Suppl 1:S22–42. 15736820 10.1080/09540120412331315303

[28] Ricci A, Bronzetti E, Mannino F, Mignini F, Morosetti C, Tayebati SK, et al Dopamine receptors in human platelets. Naunyn Schmiedebergs Arch Pharmacol. 2001;363(4):376–82. 11330330 10.1007/s002100000339

[29] Frankhauser P, Grimmer Y, Bugert P, Deuschle M, Schmidt M, Schloss P. Characterization of the neuronal dopamine transporter DAT in human blood platelets. Neurosci Lett. 2006;399(3):197–201. 16490314 10.1016/j.neulet.2006.01.062

[30] Callahan KP, Malinin AI, Atar D, Serebruany VL. Platelet activation as a universal trigger in the pathogenesis of acute coronary events after cocaine abuse. Swiss Med Wkly. 2001;131(33–34):487–9. 11683077 10.4414/smw.2001.09756

[31] Heesch CM, Wilhelm CR, Ristich J, Adnane J, Bontempo FA, Wagner WR. Cocaine activates platelets and increases the formation of circulating platelet containing microaggregates in humans. Heart. 2000;83(6):688–95. 10814631 10.1136/heart.83.6.688 PMC1760877

[32] Kugelmass AD, Oda A, Monahan K, Cabral C, Ware JA. Activation of human platelets by cocaine. Circulation. 1993;88(3):876–83. 7689042 10.1161/01.cir.88.3.876

[33] Rinder HM, Ault KA, Jatlow PI, Kosten TR, Smith BR. Platelet alpha-granule release in cocaine users. Circulation. 1994;90(3):1162–7. 7522132 10.1161/01.cir.90.3.1162

[34] Kiebala M, Maggirwar SB. Ibudilast, a pharmacologic phosphodiesterase inhibitor, prevents human immunodeficiency virus-1 Tat-mediated activation of microglial cells. PLoS One. 2011;6(4):e18633 10.1371/journal.pone.0018633 21494611 PMC3072977

[35] Kutner RH, Zhang XY, Reiser J. Production, concentration and titration of pseudotyped HIV-1-based lentiviral vectors. Nat Protoc. 2009;4(4):495–505. 10.1038/nprot.2009.22 19300443

[36] O'Brien JJ, Spinelli SL, Tober J, Blumberg N, Francis CW, Taubman MB, et al 15-deoxy-delta12,14-PGJ2 enhances platelet production from megakaryocytes. Blood. 2008;112(10):4051–60. 10.1182/blood-2008-05-158535 18755987 PMC2581982

[37] Davidson DC, Schifitto G, Maggirwar SB. Valproic acid inhibits the release of soluble CD40L induced by non-nucleoside reverse transcriptase inhibitors in human immunodeficiency virus infected individuals. PLoS One. 2013;8(3):e59950 10.1371/journal.pone.0059950 23555843 PMC3610700

[38] Kiebala M, Skalska J, Casulo C, Brookes PS, Peterson DR, Hilchey SP, et al. Dual Targeting of the Thioredoxin and Glutathione Anti-Oxidant Systems in Malignant B-cells; A Novel Synergistic Therapeutic Approach. Exp Hematol. 2014. 10.1016/j.exphem.2014.10.004 PMC4324472 25448488

[39] Ombrello C, Block RC, Morrell CN. Our expanding view of platelet functions and its clinical implications. J Cardiovasc Transl Res. 2010;3(5):538–46. 10.1007/s12265-010-9213-7 20661787 PMC3354697

[40] Rondina MT, Weyrich AS, Zimmerman GA. Platelets as cellular effectors of inflammation in vascular diseases. Circ Res. 2013;112(11):1506–19. 10.1161/CIRCRESAHA.113.300512 23704217 PMC3738064

[41] Evans SM, Cone EJ, Henningfield JE. Arterial and venous cocaine plasma concentrations in humans: relationship to route of administration, cardiovascular effects and subjective effects. J Pharmacol Exp Ther. 1996;279(3):1345–56. 8968359

[42] Chaipan C, Soilleux EJ, Simpson P, Hofmann H, Gramberg T, Marzi A, et al DC-SIGN and CLEC-2 mediate human immunodeficiency virus type 1 capture by platelets. J Virol. 2006;80(18):8951–60. 16940507 10.1128/JVI.00136-06 PMC1563896

[43] Cognasse F, Hamzeh-Cognasse H, Berthet J, Damien P, Lucht F, Pozzetto B, et al Altered release of regulated upon activation, normal T-cell expressed and secreted protein from human, normal platelets: contribution of distinct HIV-1MN gp41 peptides. AIDS. 2009;23(15):2057–9. 10.1097/QAD.0b013e328330da65 19654498

[44] Panicot-Dubois L, Thomas GM, Furie BC, Furie B, Lombardo D, Dubois C. Bile salt-dependent lipase interacts with platelet CXCR4 and modulates thrombus formation in mice and humans. J Clin Invest. 2007;117(12):3708–19. 18037996 10.1172/JCI32655 PMC2082148

[45] Rozmyslowicz T, Majka M, Kijowski J, Murphy SL, Conover DO, Poncz M, et al Platelet- and megakaryocyte-derived microparticles transfer CXCR4 receptor to CXCR4-null cells and make them susceptible to infection by X4-HIV. AIDS. 2003;17(1):33–42. 12478067 10.1097/00002030-200301030-00006

[46] Rumbaugh JA, Steiner J, Sacktor N, Nath A. Developing neuroprotective strategies for treatment of HIV-associated neurocognitive dysfunction. Futur HIV Ther. 2008;2(3):271–80. 19774095 10.2217/17469600.2.3.271 PMC2747312

[47] Johnson TP, Patel K, Johnson KR, Maric D, Calabresi PA, Hasbun R, et al Induction of IL-17 and nonclassical T-cell activation by HIV-Tat protein. Proc Natl Acad Sci U S A. 2013;110(33):13588–93. 10.1073/pnas.1308673110 23898208 PMC3746932

[48] Mediouni S, Darque A, Baillat G, Ravaux I, Dhiver C, Tissot-Dupont H, et al Antiretroviral therapy does not block the secretion of the human immunodeficiency virus tat protein. Infect Disord Drug Targets. 2012;12(1):81–6. 22280310 10.2174/187152612798994939

[49] Wang J, Zhang W, Nardi MA, Li Z. HIV-1 Tat-induced platelet activation and release of CD154 contribute to HIV-1-associated autoimmune thrombocytopenia. J Thromb Haemost. 2011;9(3):562–73. 10.1111/j.1538-7836.2010.04168.x 21143381 PMC3050111

[50] Davidson DC, Hirschman MP, Sun A, Singh MV, Kasischke K, Maggirwar SB. Excess soluble CD40L contributes to blood brain barrier permeability in vivo: implications for HIV-associated neurocognitive disorders. PLoS One. 2012;7(12):e51793 10.1371/journal.pone.0051793 23251626 PMC3520914

[51] Ziu E, Hadden C, Li Y, Lowery CL 3rd, Singh P, Ucer SS, et al Effect of serotonin on platelet function in cocaine exposed blood. Sci Rep. 2014;4:5945 10.1038/srep05945 25091505 PMC4121605

[52] Yamamoto Y, Gaynor RB. IkappaB kinases: key regulators of the NF-kappaB pathway. Trends Biochem Sci. 2004;29(2):72–9. 15102433 10.1016/j.tibs.2003.12.003

[53] O'Dea E, Hoffmann A. The regulatory logic of the NF-kappaB signaling system. Cold Spring Harb Perspect Biol. 2010;2(1):a000216 10.1101/cshperspect.a000216 20182598 PMC2827908

[54] Perkins ND. Integrating cell-signalling pathways with NF-kappaB and IKK function. Nat Rev Mol Cell Biol. 2007;8(1):49–62. 17183360 10.1038/nrm2083

[55] Oeckinghaus A, Ghosh S. The NF-kappaB family of transcription factors and its regulation. Cold Spring Harb Perspect Biol. 2009;1(4):a000034 10.1101/cshperspect.a000034 20066092 PMC2773619

[56] Torre D, Pugliese A. Platelets and HIV-1 infection: old and new aspects. Curr HIV Res. 2008;6(5):411–8. 18855651 10.2174/157016208785861140

[57] Auerbach DJ, Lin Y, Miao H, Cimbro R, Difiore MJ, Gianolini ME, et al Identification of the platelet-derived chemokine CXCL4/PF-4 as a broad-spectrum HIV-1 inhibitor. Proc Natl Acad Sci U S A. 2012;109(24):9569–74. 10.1073/pnas.1207314109 22645343 PMC3386099

[58] Solomon Tsegaye T, Gnirss K, Rahe-Meyer N, Kiene M, Kramer-Kuhl A, Behrens G, et al Platelet activation suppresses HIV-1 infection of T cells. Retrovirology. 2013;10:48 10.1186/1742-4690-10-48 23634812 PMC3660175

[59] Hanai Y, Adachi S, Yasuda I, Takai S, Matsushima-Nishiwaki R, Kato H, et al Collagen-induced p38 MAP kinase activation is a biomarker of platelet hyper-aggregation in patients with diabetes mellitus. Life Sci. 2009;85(9–10):386–94. 10.1016/j.lfs.2009.09.004 19631227

[60] Coulon L, Calzada C, Moulin P, Vericel E, Lagarde M. Activation of p38 mitogen-activated protein kinase/cytosolic phospholipase A2 cascade in hydroperoxide-stressed platelets. Free Radic Biol Med. 2003;35(6):616–25. 12957654 10.1016/s0891-5849(03)00386-1

