# Supplementary figures and images for: Platelet Activation in Human Immunodeficiency Virus Type-1 Patients Is Not Altered with Cocaine Abuse
Source: PLoS One. 2015 Jun 15;10(6):e0130061. doi: 10.1371/journal.pone.0130061 (PMC4467977; doi:10.1371/journal.pone.0130061)

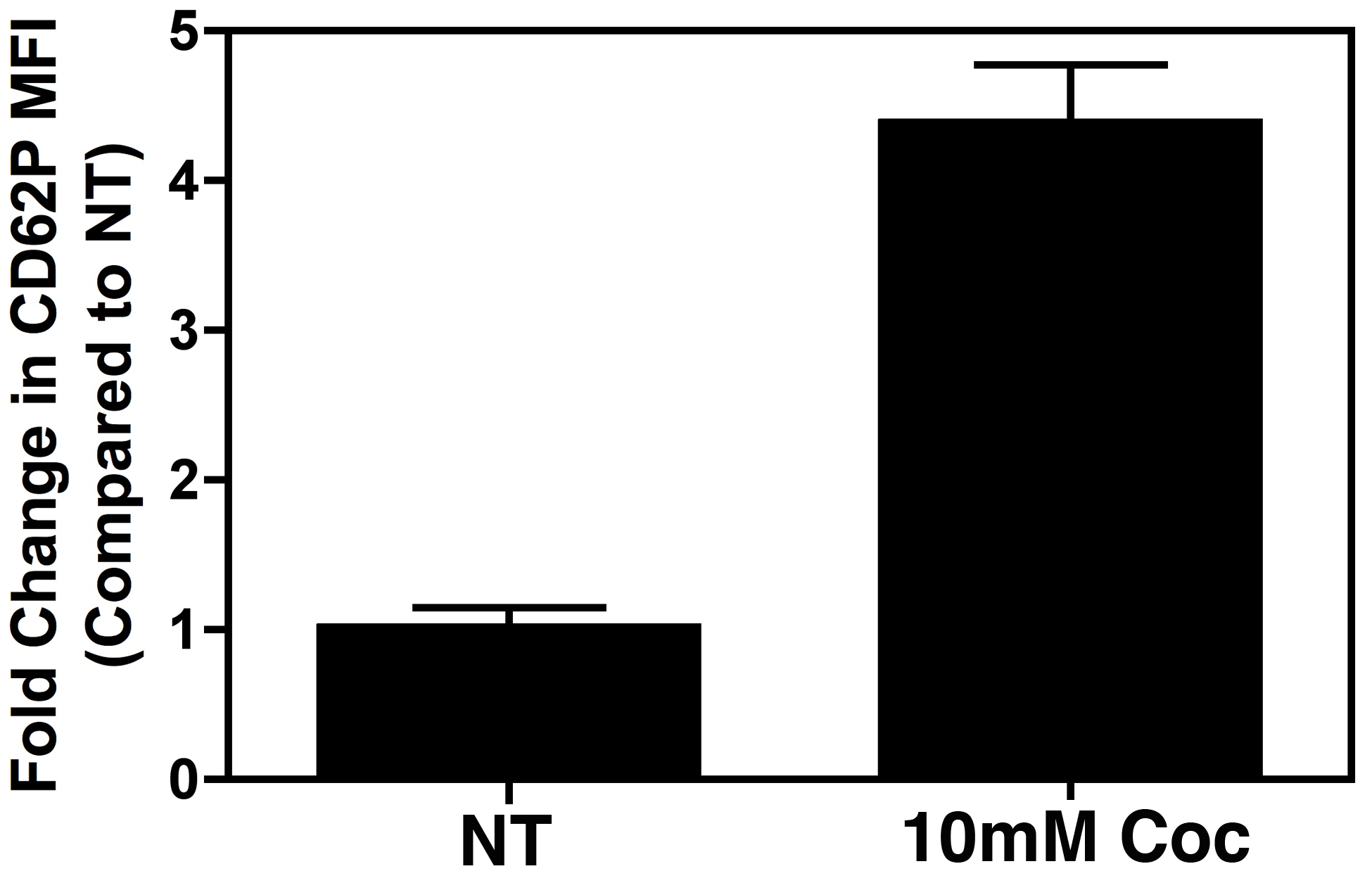

Supplement: S1 Fig — Whole blood from healthy subjects (2 donors) was left untreated (NT) or was treated with 10mM cocaine for 30 minutes, following which CD62P expression was assessed as a marker of platelet activation via flow cytometry. Treatment with this excessive amount of cocaine did result in increased platelet activation. Data are represented as fold change in CD62P MFI as compared to NT samples and are shown as mean ± SEM. (TIF) [file pone.0130061.s001.tif]

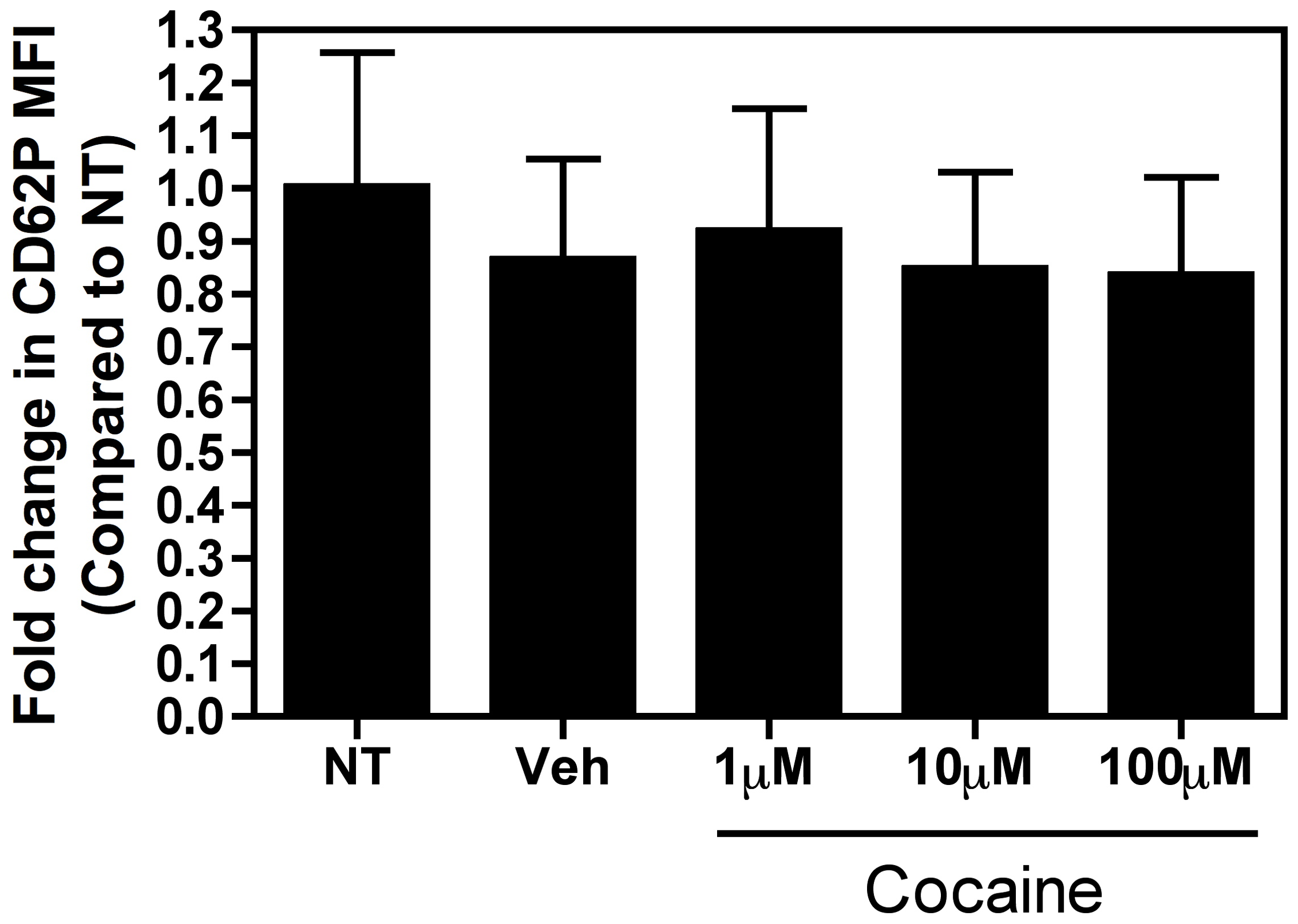

Supplement: S2 Fig — Isolated platelets from healthy subjects (4 donors) were left untreated (NT) or were treated with the indicated concentrations of cocaine for 30 minutes, following which CD62P expression was assessed as a marker of platelet activation via flow cytometry. Treatment with cocaine did not result in increased platelet activation. Treatment with 1 μL dH2O for 30 minutes was used as a vehicle (Veh) control. Data are represented as fold change in CD62P MFI as compared to NT samples and are shown as mean ± SEM. (TIF) [file pone.0130061.s002.tif]

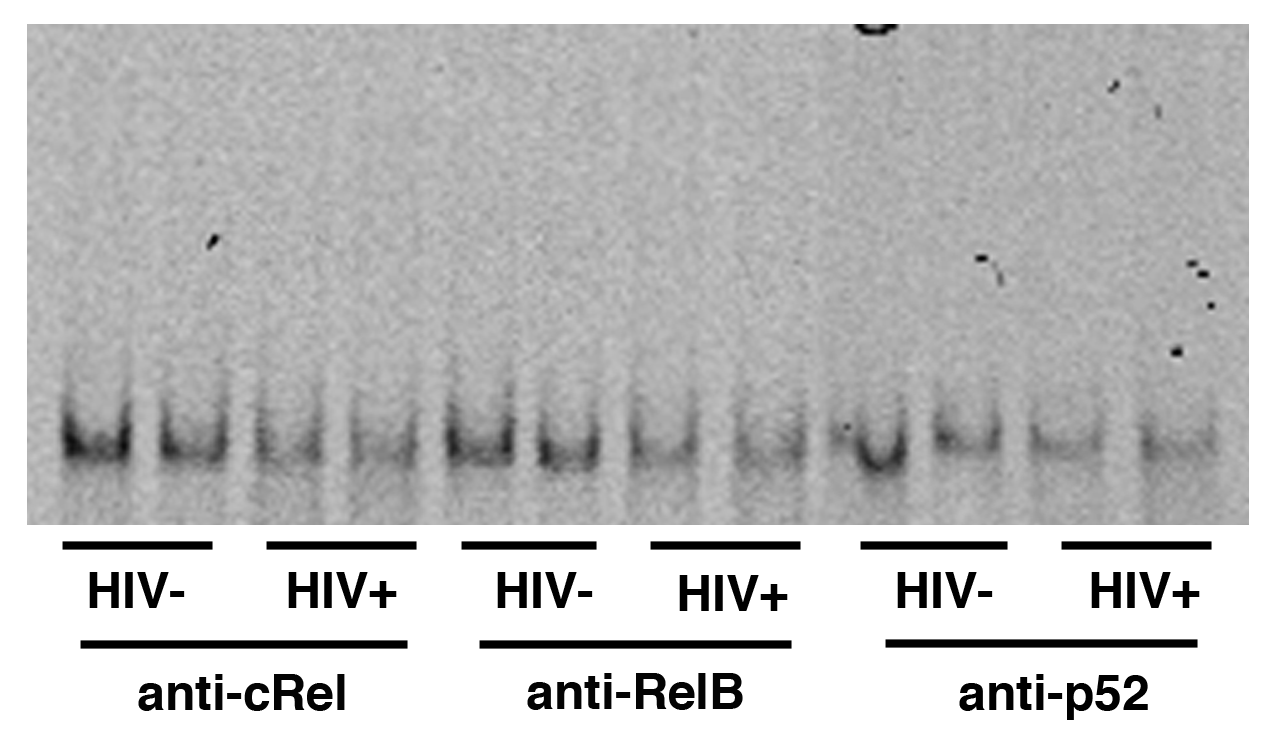

Supplement: S3 Fig — Platelet lysates collected from HIV-negative subjects (HIV-, N = 2) and HIV-positive subjects (HIV+, N = 2) were subjected to electrophoretic mobility shift assays followed by supershift with anti-cRel, anti-RelB, and anti-p52 antibodies. These antibodies did not alter the mobility of bands, suggesting that these molecules are not present in NF-κB/DNA complexes in platelets. There were also no apparent differences between HIV- and HIV+ samples. (TIF) [file pone.0130061.s003.tif]
